# Genome-Wide Association and Meta-Analysis Identify Candidate Genes for Sperm Freezability in Duroc and Yorkshire Boars

**DOI:** 10.3390/ijms27146506

**Published:** 2026-07-22

**Authors:** Siwen Wu, Jian He, Qianxi Liang, Xuehua Li, Zhuoda Lu, Zhili Li, Hui Ji, Yao Feng, Zhanwei Zhuang, Yunxiang Zhao

**Affiliations:** 1 China (Guangxi)-Vietnam Joint Laboratory for Digitalized Pig Farming, College of Animal Science and Technology, Guangxi University, Nanning 530004, China; siwenwu123@163.com (S.W.); liangqx314@163.com (Q.L.); xuehuali1998@163.com (X.L.); heyimzhuodalu@163.com (Z.L.); 2318301015@st.gxu.edu.cn (H.J.); fengyao077@163.com (Y.F.); 2 College of Animal Science and Technology, Foshan University, Foshan 528000, China; hejiansg2026@163.com (J.H.); pinganzhili@163.com (Z.L.)

**Keywords:** boar sperm freezability, heritability, GWAS, meta-analysis, candidate genes

## Abstract

Boar sperm freezability (SF) is an economically important trait that influences reproductive efficiency and genetic improvement in pigs. However, its genetic basis remains poorly understood. In this study, semen samples from 382 Duroc and 151 Yorkshire boars were evaluated for sperm motility and recovery rate, and all individuals were genotyped using an 80K SNP array. Within-breed GWAS were first conducted, followed by a meta-analysis integrating the GWAS results from both boars. Individuals were classified into GSF and PSF groups based on sperm recovery rate for subsequent selection signature analysis. The results showed that Duroc boars exhibited significantly higher SF than Yorkshire boars. Heritability estimates for SF were moderate, with values of 0.35 in Duroc and 0.30 in Yorkshire. GWAS identified 10 significant SNPs in Duroc and 33 in Yorkshire associated with SF. Meta-analysis further detected 12 significant SNPs, annotated to candidate genes such as CCDC181, PARN, SOX9, and NCKAP5L. Association analysis identified ten representative variants significantly correlated with sperm recovery rate, with variants in PARN and MAP2K6 showing strong additive effects. Selection signature analysis revealed multiple genomic regions under differential selection between GSF and PSF groups, identifying several candidate genes associated with SF. Functional enrichment analysis indicated that these genes are mainly involved in spermatogenesis, flagellar motility, cellular stress response, and cold adaptation pathways. Overall, this study provides novel insights into the genetic architecture of boar SF and identifies potential molecular markers for genetic improvement and functional genomic studies in pigs.

## 1. Introduction

Pigs are an important agricultural species that provide a major source of meat for humans and also serve as valuable biomedical models for studying human development, diseases, and organ xenotransplantation, owing to their similarities to humans in anatomical structure, physiology, and immunology [1,2,3,4,5]. Boar sperm freezability (SF) has significant economic value, as cryopreserved semen enables the widespread dissemination of superior genetic resources, enhances biosecurity compared with live animal transport, and reduces breeding costs through long-term storage [6,7,8,9]. The boar SF-associated variation and genes could also be referenced in human or other animal sperm freezability investigations.

However, boar sperm are highly sensitive to cryopreservation due to their unique membrane composition and susceptibility to low temperatures. During freezing and thawing, sperm are prone to cold shock, ice crystal formation, and reactive oxygen species (ROS)-induced oxidative damage [10]. As a result, post-thaw sperm motility is significantly reduced compared with fresh semen, leading to decreased conception rates and litter sizes [8,11,12]. Consequently, the use of frozen semen in pig production remains extremely limited, accounting for less than 0.1% of total applications [6], which severely restricts the dissemination of superior genetic resources. Notably, substantial variation in post-thaw quality of boar sperm has been observed among breeds and individuals under the same cryopreservation conditions [13]. Notably, the distinct selective histories of different pig breeds—Duroc as a terminal sire line selected for meat production traits and Yorkshire as a maternal line selected for reproductive performance—may have inadvertently shaped genomic regions controlling cellular membrane integrity, oxidative stress responses, and cold tolerance, all of which are relevant to sperm cryopreservation. These differential selection pressures provide a unique opportunity to identify genomic signatures associated with SF through population-level comparisons. Therefore, elucidating the genetic basis of SF is essential for improving cryopreservation efficiency and enhancing the application of frozen semen in pig production. Increasing evidence suggests that genetic factors, rather than environmental conditions such as freezing protocols or cryoprotectants, play a major role in determining this variation [7,12,14,15,16]. This phenomenon, referred to as SF, is now recognized as an important trait [7,12,17,18]. Therefore, elucidating the genetic basis of SF is essential for improving cryopreservation efficiency and enhancing the application of frozen semen in pig production.

Genome-wide association study (GWAS) has become a mainstream tool for identifying genetic variants associated with economically important traits in livestock and poultry. In boars, previous genomic studies have detected multiple SNPs and candidate genes associated with conventional semen quality traits, including semen volume, sperm concentration, motility, total sperm count, and sperm morphological abnormalities [19,20,21,22,23,24], indicating that these traits are under genetic control. However, the genetic basis of boar SF remains poorly understood. Recent studies have begun to fill this gap: whole-genome resequencing has identified candidate genes and variants associated with boar SF [12]. While another study confirmed that boar-to-boar variation is a major determinant of post-thaw quality of sperm and supported post-thaw total motility as a reliable indicator of boar SF [7]. Nevertheless, available molecular markers for boar SF remain limited, restricting the wider application of frozen semen in pig breeding. However, SF is a complex trait influenced by both breed-specific and shared genetic determinants. A multi-breed approach is essential to distinguish population-specific signals from evolutionarily conserved loci that are more likely to have functional relevance across genetic backgrounds. Therefore, further studies are needed to elucidate the genetic architecture of SF and identify robust molecular markers for genetic improvement.

To further elucidate the genetic basis of boar SF, this study conducted genome-wide association analyses using two commercial pig breeds, Duroc and Yorkshire. Duroc and Yorkshire were selected for this study because they represent two of the most widely used commercial pig breeds in global pork production, with distinct genetic backgrounds and documented differences in semen cryotolerance [12,15]. Duroc boars are generally known for superior post-thaw semen quality compared with Yorkshire, making them an ideal pair for cross-breed genetic comparison. This design allows us to identify both breed-specific and shared genetic determinants of SF, thereby improving the generalizability of our findings. A total of 382 Duroc and 151 Yorkshire boars were genotyped using an 80K SNP array, and SF was evaluated based on post-thaw sperm motility. Within-breed GWAS were first performed, followed by a meta-analysis integrating the results from both breeds to improve the detection power of associated loci. In addition, selection signature analyses based on *F_ST_* and *θ_π_* were applied to identify genomic regions under differential selection between groups with good sperm freezability (GSF) or poor sperm freezability (PSF). This study aimed to identify significant SNPs, candidate genes, and key biological pathways associated with boar SF, thereby providing new insights into its genetic architecture and offering potential molecular markers for genetic improvement and cryopreservation efficiency in pigs.

## 2. Results

### 2.1. Assessment of Boar Sperm Freezability

**Figure 1:**
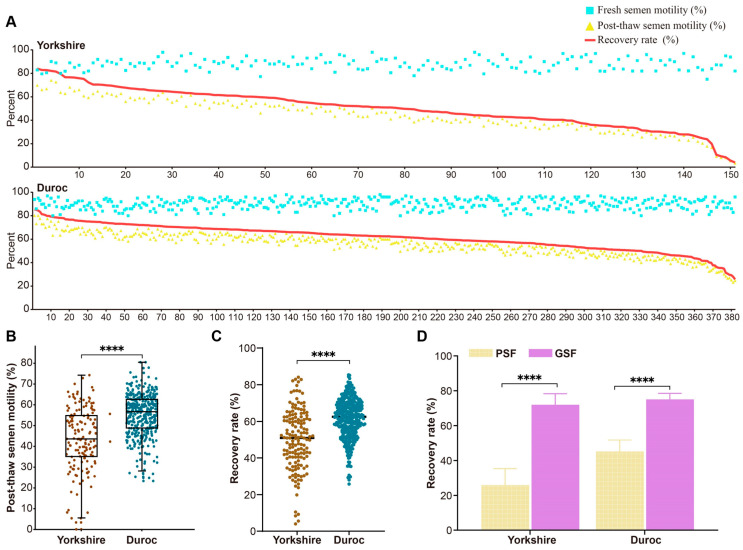
Sperm motility and recovery rate in Duroc and Yorkshire boars. (**A**) Fresh sperm motility, post-thaw sperm motility, and recovery rate in Duroc and Yorkshire boars. (**B**,**C**) Comparison of post-thaw sperm motility and recovery rate between Duroc and Yorkshire boars. (**D**) Comparison of recovery rate between GSF and PSF groups in Duroc and Yorkshire populations. **** indicates *p* < 0.0001.

Comparison of fresh and post-thaw sperm motility and recovery rate revealed a marked decline in sperm motility following cryopreservation (Figure 1A and Supplementary Table S1). Substantial phenotypic variation in SF was observed among individuals, with recovery rate ranging from 25.73% to 85.32% in Duroc and from 4.02% to 84.10% in Yorkshire populations (Supplementary Table S1). The coefficients of variation were 19% in Duroc and 32% in Yorkshire (Supplementary Table S2), indicating substantial phenotypic variability in SF among individuals.

The heritability (h^2^) of SF was estimated using a genomic relationship matrix. The heritability estimate for SF was 0.35 ± 0.11 in Duroc and 0.30 ± 0.23 in Yorkshire populations (Supplementary Table S2). These findings indicate that SF is a moderately heritable trait and may be improved through genetic selection. In addition, post-thaw sperm motility and recovery rate were significantly higher (*p* < 0.0001) in Duroc than in Yorkshire boars (Figure 1B,C), indicating substantial differences in SF between breeds. While the Duroc estimate is reasonably precise, the Yorkshire estimate carries a relatively large standard error, reflecting the smaller sample size (*n* = 151) and resulting in a wide confidence interval. Nonetheless, both estimates fall within the low-to-moderate range previously reported for boar SF [25], suggesting that SF is under genetic control and may be improved through selection.

Given the considerable variation in SF both within and between breeds, two complementary approaches were applied to identify candidate genes associated with boar SF. First, all 533 boars with semen phenotypes were genotyped using the commercial Porcine 80K SNP array, and GWAS and Meta-analysis were performed to detect genomic loci associated with SF. Second, individuals were ranked according to the Z-score of sperm recovery rate (Supplementary Table S1), and the GSF and PSF groups were defined as described in Section 4.3. The recovery rate in the GSF group was significantly higher (*p* < 0.0001) than that in the PSF group (Figure 1D). Selection signature analyses based on *F_ST_* and nucleotide diversity (*θ_π_*) were subsequently performed to identify genomic regions associated with SF.

### 2.2. Genetic Parameters and Population Structure

**Figure 2:**
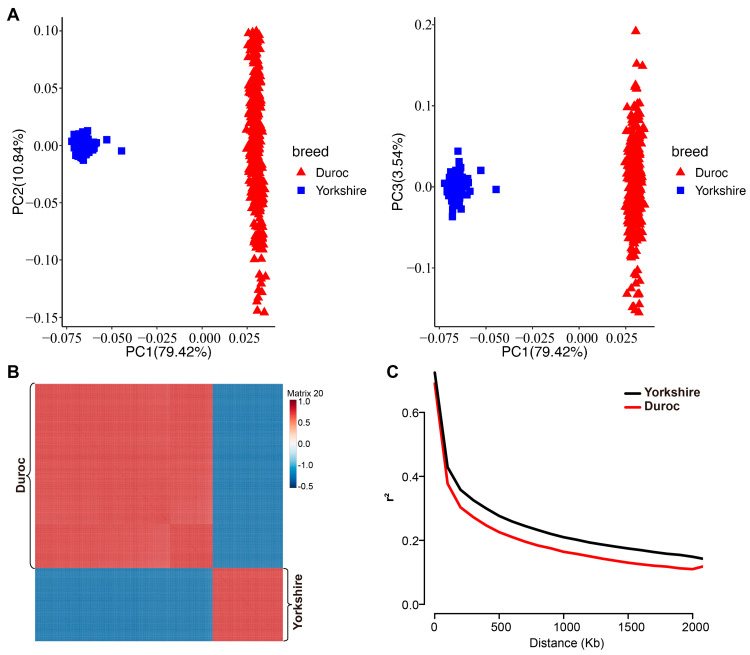
Population structure and linkage disequilibrium analysis of Duroc and Yorkshire boars. (**A**) Principal component analysis (PCA) based on genome-wide SNP data showing genetic differentiation between Duroc and Yorkshire populations. (**B**) Identity-by-state (IBS) distance heatmap illustrating genetic relationships among individuals from the two breeds. (**C**) Linkage disequilibrium (LD) decay patterns in Duroc and Yorkshire populations estimated using PopLDdecay.

After quality control of the 80K SNP genotyping data from 382 Duroc and 151 Yorkshire boars, 80,262 SNPs in Duroc and 76,128 SNPs in Yorkshire were retained for subsequent analyses (Supplementary Figure S1). Principal component analysis (PCA) clearly separated Duroc and Yorkshire populations (Figure 2A). PC1, PC2, and PC3 explained 79.42%, 10.84%, and 3.54% of the genetic variation, respectively. The first five principal components explained over 90% of the total variation and were included as covariates in the GWAS model. Identity-by-state (IBS) distance analysis showed clear clustering patterns consistent with breed classification (Figure 2B).

Linkage disequilibrium (LD) decay analysis revealed a slightly slower LD decay in Yorkshire compared with Duroc (Figure 2C). LD approached equilibrium at approximately 1 Mb in both populations; therefore, a 500 kb flanking region upstream and downstream of significant SNPs was used to define candidate gene intervals.

### 2.3. Genome-Wide Association Analysis the Boar Population

**Figure 3:**
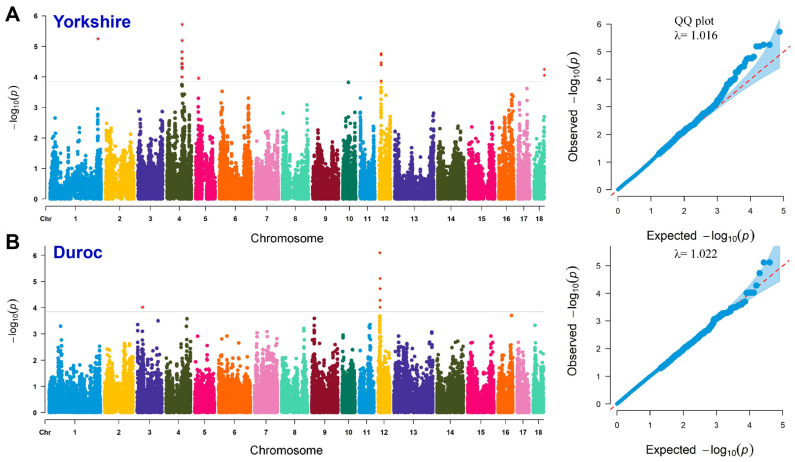
Manhattan plot and QQ plot of the Yorkshire boars (**A**) and Duroc boars (**B**). In Manhattan plots, each dot represents a SNP, with different colors indicating different chromosomes; the horizontal dashed line indicates the genome-wide significance threshold (*P* = 1.0 × 10^−5^). QQ plots show minimal deviation from the expected distribution (λ = 1.022 and 1.016 for Yorkshire and Duroc, respectively), indicating adequate control of population stratification and low inflation of false-positive signals.

Genome-wide association analysis identified 33 SNPs that were significantly associated with SF (*p* < 1.42 × 10^−4^) in the Yorkshire population, distributed across chromosomes 1, 4, 5, 12, and 18 (Figure 3A). These loci were mapped to 4 candidate genes, including *CCDC181*, *DHH*, *NCKAP5L*, and *MAP2K6* (Supplementary Table S3). In the Duroc population, GWAS identified 10 SNPs significantly associated with SF (*p* < 9.64 × 10^−5^), with 4 SNPs located on chromosome 3 and 6 SNPs on chromosome 12 (Figure 3B). All 4 SNPs on chromosome 3 were annotated to *PARN*, while all 6 SNPs on chromosome 12 were annotated to *SOX9* (Supplementary Table S4).

To further identify loci consistently associated with SF across breeds, a meta-analysis was performed by integrating GWAS results from the Duroc and Yorkshire populations. A total of 12 SNPs were significantly associated with SF (Supplementary Table S5), of which 8 overlapped with loci detected in the breed-specific GWAS, indicating shared genetic components underlying sperm cryotolerance. In addition, 4 novel SNPs (rs318643485, yz_rs346137756, rs81438940, and yz_12_9412094) were identified exclusively through meta-analysis, suggesting additional loci with moderate but consistent effects across populations.

Collectively, meta-analysis and single-breed GWAS identified several genomic regions associated with SF, including *PARN*, *SOX9*, *CCDC181*, *DHH*, *NCKAP5L*, and *MAP2K6* (Supplementary Tables S5–S7). To further evaluate the effects of these loci, association analysis between genotypes and sperm recovery rate was performed using a general linear model. Ten variants were significantly associated with sperm recovery rate (Table 1, Tables S6 and S7). Among these loci, variants located within *MAP2K6* and *PARN* exhibited relatively large additive effects (>9), indicating strong allelic dosage effects on sperm cryotolerance. Furthermore, multiple significant SNPs were clustered within the *PARN* region on chromosome 3, forming a consistent association signal supported by both GWAS and meta-analysis.

### 2.4. Selection Signature Analysis of Boar Population

To further identify genomic regions associated with SF, selection signature analysis was performed between the GSF and PSF groups within each breed (group classification shown in Figure 1D and Section 4.3). Genome-wide population differentiation was evaluated using fixation index (*F_ST_*) statistics. The overall genetic differentiation between GSF and PSF groups was low in both populations, with mean *F_ST_* values of 0.0034 in Yorkshire and 0.0035 in Duroc (Supplementary Figure S2).

Using the top 1% of *F_ST_* values as the threshold, 422 candidate selection regions (*F_ST_* > 0.082) were identified in Yorkshire boars, distributed across chromosomes 1, 2, 3, 4, 6, 12, 15, 16, and 18, with the strongest selection signals detected on chromosomes 4 and 16 (Supplementary Figure S2A). In the Duroc population, 387 candidate regions (*F_ST_* > 0.043) were detected across chromosomes 1, 2, 3, 4, 5, 6, 8, 9, 11, 12, 14, 15, and 16, with the most prominent signals located on chromosomes 3 and 11 (Supplementary Figure S2B). The difference in *F_ST_* thresholds between Yorkshire (0.082) and Duroc (0.043) reflects breed-specific genetic backgrounds and the varying levels of genomic differentiation between GSF and PSF groups within each population.

**Figure 4:**
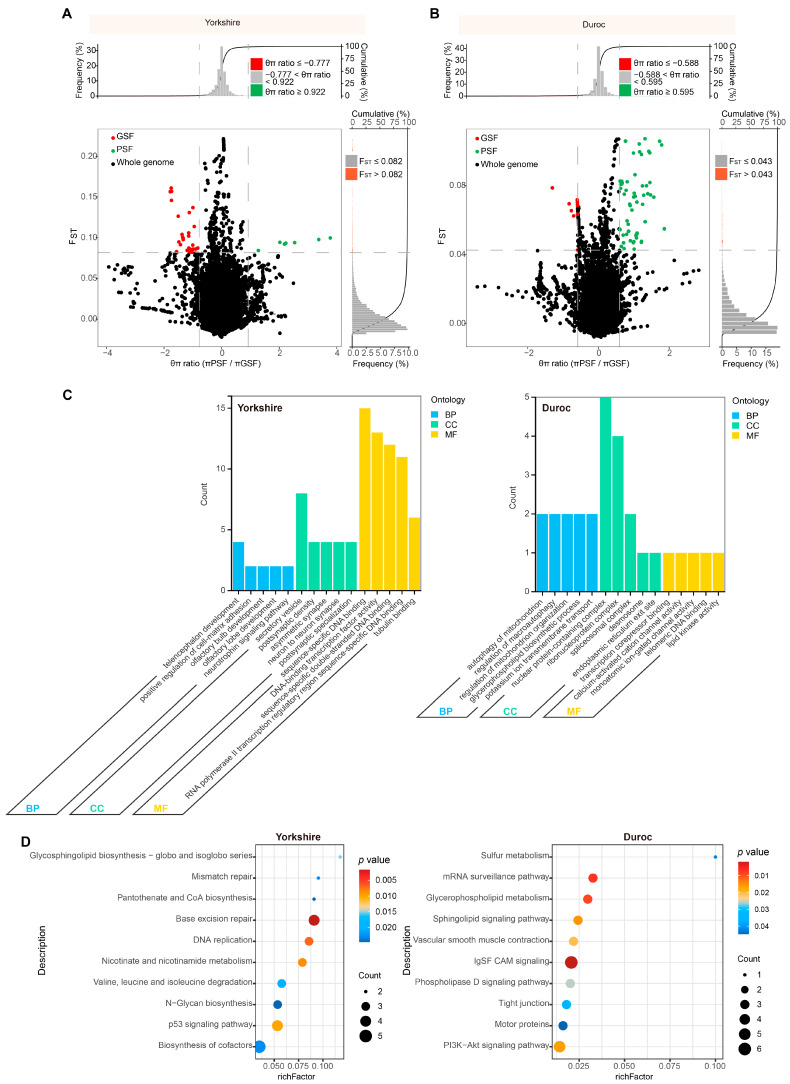
Selection signature analysis and functional enrichment of candidate genes associated with SF in Duroc and Yorkshire boars. (**A**) Genome-wide distribution of selection signals identified by combined *F_ST_* and *θπ* analysis between GSF and PSF groups in Yorkshire boars. (**B**) Genome-wide distribution of selection signals identified by combined *F_ST_* and *θπ* analysis between GSF and PSF groups in Duroc boars. In both panels (**A**,**B**), dashed vertical lines indicate the upper and lower top 1% thresholds for θπ ratio, and dashed horizontal lines indicate the top 1% thresholds for FST. Regions falling beyond these cutoffs were considered candidate selection regions. (**C**) Top5 GO terms of candidate genes identified from *F_ST_* and *θπ* analysis in Yorkshire (left) and Duroc (right) populations. (**D**) Top 10 KEGG pathways of candidate genes identified from *F_ST_* and *θπ* analysis in Yorkshire (**left**) and Duroc (**right**) populations.

To increase the robustness of selection signal detection, nucleotide diversity (*θ_π_*) analysis was further integrated with *F_ST_* results. In Yorkshire boars, the nucleotide diversity (*θ_π_*) values were 0.481 and 0.479 for the GSF and PSF groups, respectively, with corresponding *θ_π_* ratio values of 0.922 and −0.777 (Figure 4A). Based on the top 1% thresholds, 422 candidate regions were identified using *F_ST_* and 397 candidate regions using *θ_π_*. The combined *F_ST_* and *θ_π_* analysis identified 153 overlapping genomic regions, which were annotated to 266 genes (Supplementary Figure S3A). Similarly, in the Duroc population, *θ_π_* were 0.481 and 0.488 for the GSF and PSF groups, respectively, with *θ_π_* ratio values of 0.595 and −0.588 (Figure 4B). Using the top 1% thresholds, 387 candidate regions were identified based on *F_ST_* and 443 regions based on *θ_π_*. A total of 81 overlapping regions were identified by combined *F_ST_* and *θ_π_* analysis, containing 77 annotated genes (Supplementary Figure S3B).

Functional enrichment analysis of the identified genes revealed several biological processes and pathways associated with sperm cryotolerance (Figure 4C,D). Based on functional annotation, PigQTL database information, and previous studies, four candidate genes related to SF were identified in Duroc boars (*RDH5*, *PSMA8*, *TPGS2*, and *SSX2IP*), while sixteen candidate genes were identified in Yorkshire boars (*PRXL2C*, *PLCL2*, *SUN3*, *CCDC181*, *MAEL*, *ELSPBP1*, *IZUMO1*, *PPP1R15A*, *PIH1D1*, *SNORD34*, *SNORD35A*, *TSKS*, *IZUMO2*, *CC2D2A*, *ACRV1*, and *DDX25*). These genes are primarily involved in spermatogenesis, ciliogenesis, flagellar motility, germ cell development, cellular stress response, and adaptive regulation under low-temperature conditions.

## 3. Discussion

SF is a complex quantitative trait influenced by both genetic and environmental factors [9,12]. Strategies to mitigate cryoinjury in boar semen have evolved from traditional process optimization to multi-level approaches integrating extender optimization [26,27], cryoprotectant supplementation [28,29], antioxidant addition [30], and freezing protocol refinement [31]. However, improving semen cryotolerance through the genetic selection of boars with superior SF [12,15,25,32] may represent a more sustainable and efficient strategy for enhancing semen quality and reproductive performance, particularly with the increasing availability of molecular markers associated with SF.

While the 80K SNP array offers lower resolution than whole-genome resequencing, the integration of multiple selection signatures (e.g., *F_ST_*, *θπ*, and XP-EHH) in a meta-analysis helps compensate for this limitation by cross-validating candidate regions and reducing false positives. In the present study, we combined GWAS, meta-analysis, and selection signature analysis to systematically investigate the genetic basis underlying variation in boar sperm cryotolerance. Phenotypic results showed that Duroc boars exhibited significantly higher post-thaw motility and recovery rates than Yorkshire boars, indicating clear breed-specific differences in resistance to cryodamage. This finding is consistent with previous reports suggesting that sperm cryotolerance varies among pig breeds and is partially genetically determined [12,15,25]. Previous studies have estimated the heritability of SF to range from low to moderate levels [25]. In agreement with these findings, the moderate heritability estimates obtained in this study (0.35 in Duroc and 0.30 in Yorkshire) further support that SF is under genetic control and has considerable potential for improvement through marker-assisted or genomic selection. In addition, the heritability estimate for Yorkshire (0.30 ± 0.23) should be interpreted with caution due to its large standard error, which is a direct consequence of the modest sample size (*n* = 151) available for this breed. This imprecision means the true heritability could range from near-zero to moderately high, and the estimate may not be robust enough for precise genomic prediction. Future studies with larger Yorkshire cohorts will be needed to obtain more reliable variance component estimates and to validate the breed-specific genetic parameters reported here.

The further genome-wide association analysis identified several genomic regions significantly associated with SF, including *PARN* and *SOX9* in Duroc, and *CCDC181*, *DHH*, *NCKAP5L*, and *MAP2K6* in Yorkshire populations. Meta-analysis further confirmed shared genetic components across breeds and identified additional loci that were not detected in single-breed GWAS, demonstrating the advantage of combining multi-population datasets to improve detection power for complex traits. Several candidate genes identified in this study have known biological functions related to spermatogenesis and sperm structural integrity. For example, *SOX9* plays an essential regulatory role in Sertoli cell differentiation and testicular development [33,34,35], which are critical for sperm production and maturation. *CCDC181* is involved in cilia and flagella formation, which directly affects sperm motility [36,37]. *DHH* participates in testicular development and germ cell differentiation [38,39], while *NCKAP5L* has been implicated in cytoskeletal organization, potentially influencing sperm morphology and motility stability during cryopreservation [40].

Notably, variants located within *MAP2K6* and *PARN* exhibited relatively large additive effects, suggesting a strong allelic dosage effect on sperm cryotolerance. Consistent with previous studies reporting that allelic polymorphisms can serve as predictive markers of boar SF [15], our results further support the potential utility of *MAP2K6* and *PARN* variants as molecular markers for improving sperm cryotolerance through genetic selection. *MAP2K6* encodes a key kinase in the p38 MAPK signaling pathway, which is a central mediator of cellular responses to environmental stresses, including oxidative stress, osmotic shock, and cold exposure [41,42]. During sperm cryopreservation, cells are sequentially exposed to cooling, ice crystal formation, and osmotic imbalances, all of which activate the p38 MAPK cascade. Activation of this pathway can trigger either pro-survival adaptive responses or apoptotic cell death, depending on the magnitude and duration of the stress [42]. Polymorphisms affecting *MAP2K6* expression or kinase activity may therefore shift this balance, influencing the ability of spermatozoa to withstand cryoinjury. Our finding that *MAP2K6* SNPs exhibited relatively large additive effects on sperm recovery rate supports this hypothesis and suggests that *MAP2K6* may serve as a promising candidate for marker-assisted selection to improve boar SF. Future functional studies using gene editing or pharmacological modulation in sperm models will be necessary to validate the causal role of *MAP2K6* in cryosurvival. Functional enrichment analysis further supported the involvement of *MAP2K6* in biological processes relevant to sperm cryotolerance. KEGG pathway analysis indicated that *MAP2K6* participates in the GnRH signaling pathway and the MAPK signaling pathway, both of which are closely associated with gonadal development, hormonal regulation, and cellular stress adaptation. GO enrichment results revealed that *MAP2K6*, together with *DHH* and *CCDC181*, was enriched in functional categories related to Leydig cell differentiation, MAP kinase activity, MAPK cascade, spermatid development, microtubule binding, and protein serine/threonine kinase activity. Cryopreservation induces multiple stressors in sperm cells, including oxidative stress, osmotic imbalance, and membrane damage, all of which can impair sperm motility and viability. Therefore, genetic variants involved in stress-response pathways may represent important determinants of sperm survival following freezing and thawing.

Selection signature analysis further revealed genomic regions showing significant differentiation between GSF and PSF groups. Functional enrichment analysis indicated that these genes were mainly involved in spermatogenesis, cilium assembly, microtubule organization, and cellular stress response pathways, which are closely related to sperm motility, flagellar structure, and resistance to cryoinjury. Several genes identified through selection signature analysis, such as *SUN3* [43,44], *CCDC181* [36,37], *ACRV1* [45] and *ELSPBP1* [46,47], are known to participate in sperm development, acrosome reaction, and flagellar function, highlighting the importance of sperm structural integrity and fertilization capacity in determining freezability.

Compared with previous studies [12] based on whole-genome resequencing, which identified genes such as *SPAG9*, *LRBA*, and *PPP3CA* associated with sperm recovery rate, the present study identified both previously reported and novel candidate genes, including *MAP2K6*, *NCKAP5L*, and *RDH5*, through the integration of SNP array-based GWAS and selection signature analysis. These findings expand the current understanding of the genetic architecture of boar SF and suggest that stress-response pathways, cytoskeleton organization, and flagellar assembly may represent key biological mechanisms influencing sperm cryotolerance.

One notable observation is that the Yorkshire population (*n* = 151) yielded 33 significant SNPs, compared with only 10 in the larger Duroc cohort (*n* = 382). While this could partly reflect genuine breed-specific genetic architecture, the higher number of associations in the smaller cohort raises the possibility of false-positive findings due to reduced statistical power and increased sampling variance. To mitigate this risk, we applied FDR correction and cross-validated the Yorkshire-specific SNPs using meta-analysis, which integrates effect estimates from both breeds and retains only those with consistent direction and magnitude of effect. The fact that 8 of the 12 meta-analysis-significant SNPs overlapped with breed-specific GWAS results supports the robustness of the shared signals. Nevertheless, we caution that some Yorkshire-specific associations may be population-specific or spurious, and recommend validation in independent cohorts before their application in breeding programs. Overall, the integration of GWAS, meta-analysis, and selection signature analyses consistently highlighted several genomic regions potentially associated with sperm cryotolerance. Subsequent association analysis further identified ten representative variants, among which two SNPs (rs325752048 and rs337985165) located within the *MAP2K6* gene showed the strongest genetic effects, highlighting the potential role of MAPK-related pathways in regulating sperm resistance to cryoinjury.

In summary, this study revealed both shared and breed-specific genetic architectures underlying boar SF. Duroc boars, which exhibited superior post-thaw semen quality, showed significant associations primarily within the *PARN* and *SOX9* loci, both of which are involved in testicular development and spermatogenesis. In contrast, Yorkshire boars, with lower overall SF, displayed a broader set of associations, including *CCDC181, DHH*, *NCKAP5L*, and *MAP2K6*, with the latter two implicating cytoskeletal organization and stress-response pathways. These breed-specific differences likely reflect the divergent selection histories of Duroc (terminal sire, selected for meat production) and Yorkshire (maternal line, selected for reproductive traits), which may have inadvertently shaped genomic regions controlling membrane integrity, oxidative stress responses, and cold tolerance. Notably, the cross-breed meta-analysis identified shared signals in *CCDC181* and *MAP2K6*, suggesting that certain genetic determinants of SF are conserved across breeds, while others are population-specific. These findings highlight the importance of multi-breed approaches for dissecting the genetic architecture of complex traits and underscore the need for breed-specific strategies in genomic selection for improved sperm cryotolerance. Future functional studies and validation in independent cohorts will be essential to confirm the causal roles of these candidate genes and to translate them into practical breeding tools.

## 4. Materials and Methods

### 4.1. Animals and Semen Collection

A total of 533 boars, including 382 Duroc and 151 Yorkshire boars, were used in this study; the age of each boar is listed in Supplementary Table S1. All the boars were healthy, fertile, and mature, and were individually housed at the Yangxiang original breeding pig farm of Guangxi Yangxiang Group Co., Ltd. (Guigang, Guangxi, China). All experimental procedures were approved by Guangxi University (No. GXU-2026-018). Fresh semen was collected using the gloved-hand method, and only the sperm-rich fraction (SRF) was collected for each ejaculate to minimize seminal plasma contamination and ensure consistent sperm quality. One ejaculate per boar was collected and used for cryopreservation and phenotyping. Semen samples were transported to the production laboratory using a pneumatic transfer system. Fresh semen quality was assessed immediately after collection using a computer-assisted sperm analysis (CASA) system (IVOS II, Hamilton Thorne, Inc., Beverly, MA, USA). High-quality semen samples meeting the following criteria were selected for cryopreservation: sperm motility > 75%, sperm concentration ≥ 200 × 10^6^/mL, and normal morphology > 85% (abnormal morphology < 15%).

### 4.2. Sperm Processing and Cryopreservation Protocol

Cryopreservation of boar sperm was performed according to the enterprise semen cryopreservation protocol. Fresh semen samples were diluted at a 1:1 (*v*/*v*) ratio with a commercial extender (CryoPlus, Minitüb GmbH, Tiefenbach, Germany) and cooled to 17 °C for 2–3 h. The cooled semen was then centrifuged at 950× *g* for 20 min at 17 °C using a constant-temperature centrifuge (KDC-2046 Low-Speed Refrigerated Centrifuge, Zonkia Scientific Instruments Co., Ltd., Hefei, Anhui, China) to collect sperm pellets. This centrifugation condition has been routinely validated in our laboratory and the collaborating farm’s workflow, with no significant adverse effects on post-thaw sperm quality in preliminary trials. The pellets were resuspended in cooling solution I and incubated at 5 °C for 3–4 h. Subsequently, freezing solution II containing 3% glycerol and 1.5% egg yolk was added to the sperm suspension to a final concentration of 1.5 × 10^9^ sperm/mL.

The processed semen was loaded into 0.5 mL polyvinyl chloride straws using an automated filling and sealing device (Minitüb, Minitüb GmbH, Tiefenbach, Germany), and cryopreserved at a programmed rate with a controlled-rate freezer (IceCube 14S, Minitüb, Tiefenbach, Germany) using the following cooling curve: from 5 °C to −5 °C at 6 °C/min, from −5 °C to −80 °C at 40 °C/min, followed by immersion in liquid nitrogen (−196 °C) for long-term storage. After at least 24 h of storage, the frozen semen straws were thawed in a water bath at 50 °C for 16 s, transferred into tubes containing pre-warmed diluent at 33 °C, and incubated at 37 °C for 2 min. Post-thaw sperm motility was then evaluated using the CASA system (IVOS II, Hamilton Thorne, Inc., Beverly, MA, USA) as described in Section 4.1.

### 4.3. Phenotype Definition of Sperm Freezability

Sperm freezability (SF) was evaluated based on post-thaw sperm motility measured using the CASA system. SF was defined as the motility of sperm after freezing-thawing treatment. Higher post-thaw sperm motility indicates greater tolerance to cryopreservation.

SF is reflected by the post-thaw recovery rate of sperm motility. The recovery rate [12] is calculated using the following formula:

$$x_{i}=\frac{Y_{i}}{X_{i}}\times 100\%$$

The *x*_i_ is the recovery rate of the *i_th_* boar, $Y_{i}$ is the post-thaw sperm motility of the *i_th_* boar, and $X_{i}$ is the fresh sperm motility of the *i_th_* boar. To eliminate the influence of different batches, the Z-score method was applied to standardize the sperm recovery rate, as follows:

$$Z_{i}=\frac{x_{i}-\mu }{\sigma }$$

Here *Z_i_* represents the standardized Z-score of the recovery rate for the *i_th_* boar, *x*_i_ is the recovery rate of the *i_th_* boar, *μ* is the mean recovery rate of all boars in each batch, and *σ* is the standard deviation of the recovery rate of all boars in each batch. The standard deviation *σ* is calculated using:

$$\sigma =\sqrt{\frac{1}{N}}\sum_{i=1}^{N}{\left(x_{i}-\mu \right)}^{2}$$

where *σ* is the standard deviation of the recovery rate, *N* is the number of boars, *x*_i_ is the recovery rate of the *i_th_* boar, and *μ* is the mean recovery rate of all boars in each batch.

For selection signature analysis, individuals within each breed were ranked according to Z-score values. The top 20% of individuals were defined as the good sperm freezability (GSF) group (76 Duroc and 30 Yorkshire boars), while the bottom 20% were defined as the poor sperm freezability (PSF) group (76 Duroc and 30 Yorkshire boars).

### 4.4. Genotype Data Acquisition and Quality Control

Genomic DNA was extracted from ear tissue samples of Duroc and Yorkshire boars. DNA integrity was assessed by 1% agarose gel electrophoresis, and DNA quality and concentration were evaluated using a NanoDrop 2000 spectrophotometer (Thermo Scientific, Waltham, MA, USA). Qualified DNA samples were genotyped using the commercial Porcine 80K SNP array from Guangzhou Yingzi Technology Co., Ltd. (Guangzhou, China), yielding a total of 187,255 SNPs. Genotype quality control was performed using PLINK v1.9 software [48]. SNP data were first filtered, followed by genotype imputation using the Beagle software v5.4 [49] to infer missing genotypes, and a second round of quality control was subsequently conducted. The quality control criteria were as follows: (1) individuals with call rates < 90% were excluded; (2) SNPs with call rates < 90% were removed; (3) SNPs with minor allele frequency (MAF) < 0.01 were excluded; and (4) SNPs deviating from Hardy–Weinberg equilibrium (*p* < 1 × 10^−6^) were removed. After quality control, a total of 382 Duroc boars (80,262 SNPs) and 151 Yorkshire boars (76,128 SNPs) with complete genotype and phenotype data were retained for subsequent analyses.

### 4.5. Population Structure and Linkage Disequilibrium

Population structure was assessed using PLINK v1.9 [48] based on quality-controlled SNP data. Principal component analysis (PCA) was performed to evaluate genetic stratification between Duroc and Yorkshire boar populations. Identity-by-descent (IBD) distances were calculated to evaluate genetic relatedness among individuals.

Linkage disequilibrium (LD) decay was estimated using the PopLDdecay software v3.41 [50]. LD decay describes the decline in correlation coefficient (r^2^) between genetic markers as physical distance increases across the genome. LD decay patterns were calculated separately for Duroc and Yorkshire boar populations.

### 4.6. Heritability Estimation

The linear mixed model implemented in the GCTA software v1.94 [51] was used to estimate the heritability of SF in Duroc and Yorkshire populations. A genomic relationship matrix was constructed based on SNP data. The statistical model was defined as:

$$y=X\beta +g+\varepsilon ,\;var(y)=A_{g}\sigma_{g}^{2}+I\sigma_{\varepsilon }^{2}$$

where *y* represents the vector of phenotypic values of SF; *X* represents the incidence matrix of fixed effects; *β* represents the vector of fixed effects, including boar age at semen collection and collection month; g represents the random additive genetic effect of individuals; and *ε* represents the residual error. The additive genetic variance and residual variance were estimated to calculate heritability; *A_g_* represents the kinship matrix computed based on the SNPs; $\sigma_{g}^{2}$ represents the additive genetic variance of the SNPs; *I* represents the indicator matrix; and $\sigma_{\varepsilon }^{2}$ is the residual variance.

### 4.7. Genome-Wide Association Study

Genome-wide association studies were conducted separately for Duroc and Yorkshire boar populations using the univariate mixed linear model implemented in GEMMA software v0.98.5 [52]. The statistical model was defined as:

y=Wα+Xβ+μ+ε

where *y* represents the vector of SF phenotypes; *W* represents the matrix of fixed effects and covariates, including boar age, semen collection month, and the first five principal components derived from PCA; *X* represents the genotype matrix of SNP markers; *β* represents SNP effects; *μ* represents the random polygenic effect accounting for population structure; and *ε* represents residual error.

To control false positive signals caused by multiple testing, the false discovery rate (FDR) method was used to adjust significance thresholds. The genome-wide significance threshold was determined according to:

P=FDR×n/m

where *FDR* was set to 0.01, *n* represents the number of loci with *p*-values less than 0.01 in the GWAS results, and *m* represents the total number of SNPs after quality control. To control false positives due to multiple testing, the Benjamini–Hochberg false discovery rate (FDR) method was applied using the p.adjust function in R with q = 0.05. SNPs with adjusted *P* < 0.05 were considered genome-wide significant. This yielded breed-specific significance thresholds of *P* < 6.81 × 10^−5^ for Duroc and *P* < 1.02 × 10^−4^ for Yorkshire, reflecting the different numbers of SNPs retained after quality control in each population (80,262 and 76,128, respectively).

### 4.8. Meta-Analysis

Meta-analysis was performed by combining GWAS summary statistics from Duroc and Yorkshire populations using METAL software version 2011-03-25 [53]. Effect sizes and *p*-values from each breed were converted into Z-scores, and a weighted Z-test (Z-scores) was used to calculate combined association statistics:

$$Z=\frac{\Sigma_{i}\emptyset^{-1}\left(\frac{P_{i}}{2}\right)*sign\left({∆}_{i}\right)*\sqrt{N_{i}}}{\sqrt{\Sigma_{i}N_{i}}}$$

where *P_i_* represents the *p*-value for the *i_th_* population, Δ*_i_* represents the direction of the effect for the *i_th_* population, and *N_i_* is the sample size for the *i_th_* population.

### 4.9. Selection Signature Analysis

Selection signature analysis was performed to identify genomic regions potentially associated with SF differences between the GSF and PSF groups defined in Section 4.3.

Genetic differentiation between the GSF and PSF groups was evaluated using fixation index (*F_ST_*) statistics, which quantify allele frequency differences between populations. Nucleotide diversity (*θ_π_*) was calculated to assess within-population genetic variation. Both *F_ST_* and *θ_π_* statistics were calculated using VCFtools v0.1.16 [54] based on a sliding window approach with a window size of 500 kb and a step size of 50 kb across the genome.

*F_ST_* values range from 0 to 1, where larger values indicate greater genetic differentiation between populations. Nucleotide diversity (*θ_π_*) reflects the level of genetic variation within a population, with lower values suggesting potential selection pressure. The top 1% of genomic windows ranked by *F_ST_* and *θ_π_* were considered candidate regions under selection. Regions simultaneously identified by both methods were regarded as strongly selected candidate regions.

The *F_ST_* statistic was calculated as:

$$F_{ST}=\frac{H_{t}-H_{s}}{H_{t}}$$

where *H_t_* represents the total heterozygosity of the combined population, and *H_s_* represents the average heterozygosity within subpopulations.

Nucleotide diversity (*θ_π_*) was calculated as:

$$\pi =\sum_{ij}x_{i}x_{j}\pi_{ij}=2*\sum_{i=1}^{n}\sum_{j=1}^{i-1}x_{i}x_{j}\pi_{ij}$$

where *x_i_* and *x_j_* represent allele frequencies, and *π_ij_* represents the nucleotide differences between sequences.

The ratio of nucleotide diversity between populations was calculated as:

$$\theta_{\pi ratio}=\frac{\theta_{\pi A}}{\theta_{\pi B}}$$

Genomic regions simultaneously identified in the top 1% of both *F_ST_* and *θ_π_* distributions were considered candidate regions associated with SF.

### 4.10. Candidate Gene Identification and Functional Enrichment

Functional annotation of candidate genes was performed based on the Sus scrofa annotation database (org.Ss.eg.db) in R. Gene Ontology (GO) and Kyoto Encyclopedia of Genes and Genomes (KEGG) enrichment analyses were conducted using the clusterProfiler package. Gene ID mapping and annotation were implemented using AnnotationDbi, while enrichment visualization was performed using ggplot2, enrichplot, and tidyverse packages. Biological process (BP), molecular function (MF), and cellular component (CC) categories were included in GO enrichment analysis. Pathways or GO terms with *p* < 0.05 were considered statistically significant.

### 4.11. Statistical Analysis

All phenotypic data are presented as mean ± standard deviation (SD). Differences in fresh sperm motility, post-thaw sperm motility, and recovery rate between Duroc and Yorkshire, and recovery rate between the GSF and PSF groups within each breed were evaluated using an unpaired two-tailed Student’s *t*-test implemented in GraphPad Prism v10.4.2 Association analyses between genotypes and phenotypes were performed using a general linear model with the Student-Newman-Keuls test in IBM SPSS Statistics 26.0. The model [12] is as follows:

$$y_{ijkl}=\mu +G_{i}+B_{j}+P_{k}+e_{ijkl}$$

where y*_ijkl_* represents the sperm recovery rate, *μ* represents the overall population mean, *G*_i_ represents the genotypic effect (with *i* = 1, 2, 3 indicating different genotypes), *B*_j_ represents the breed effect, *P*_k_ represents the batch effect, and *e_ijkl_* represents the random error.

## 5. Conclusions

Taken together, this study systematically investigated the genetic architecture of boar sperm freezability through the integration of GWAS, meta-analysis, and selection signature analyses in Duroc and Yorkshire populations. Several candidate genes, including *MAP2K6*, *PARN*, *CCDC181*, *DHH*, *NCKAP5L*, and *SOX9*, were consistently identified across analytical approaches, suggesting the involvement of spermatogenesis-related and stress-response pathways in determining sperm cryotolerance. Notably, association analysis identified ten representative variants significantly associated with sperm recovery rate, with SNPs in *PARN* and *MAP2K6* showing relatively strong additive genetic effects. Although these genes have established roles in reproductive processes and cellular stress regulation, their direct contributions to sperm cryosurvival remain largely unclear. While our findings provide valuable insights and potential molecular markers for boar SF, further validation in independent populations and additional breeds is necessary to confirm the generalizability of these candidate genes and SNPs. Larger and more balanced multi-breed cohorts, combined with functional validation studies (e.g., gene editing or in vitro assays), will be essential to translate these genetic markers into practical tools for genomic selection and to fully elucidate the underlying biological mechanisms of sperm cryotolerance.

## Figures and Tables

**Table 1:** Ten representative variants are significantly associated with SF in boars.

Breed	SNP ID	Gene	Genotype (Counts)	Recovery Rate (100%)	Chr	*p*-Value	Length
Yorkshire	rs343874730	*CCDC181*	AA (1)	67.16 ± 0.00 ^a^	4	1.90 × 10^−6^	-279422
			AC (36)	59.96 ± 13.90 ^a^			
			CC (114)	46.81 ± 15.95 ^b^			
	rs335125992	*DHH|NCKAP5L*	AA (51)	45.50 ± 17.52 ^b^	5	1.11 × 10^−4^	-1781988
			GA (74)	51.08 ± 15.86 ^ab^			
			GG (26)	56.21 ± 13.73 ^a^			
	rs710355473	*DHH|NCKAP5L*	CC (26)	56.21 ± 13.73 ^a^	5	1.11 × 10^−4^	-1782067
			CT (74)	51.08 ± 15.86 ^ab^			
			TT (51)	45.50 ± 17.52 ^b^			
	rs325752048	*MAP2K6*	AA (22)	62.84 ± 11.50 ^a^	12	1.74 × 10^−5^	within
			AG (84)	49.97 ± 15.16 ^b^			
			GG (45)	44.05 ± 17.49 ^b^			
	rs337985165	*MAP2K6*	AA (45)	44.05 ± 17.49 ^b^	12	1.74 × 10^−5^	within
			GA (84)	49.97 ± 15.16 ^b^			
			GG (22)	62.84 ± 11.50 ^a^			
Duroc	rs322459109	*PARN*	AA (344)	62.09 ± 10.09 ^a^	3	9.48 × 10^−5^	within
			GA (37)	54.82 ± 13.79 ^b^			
			GG (1)	37.60 ± NA ^b^			
	rs333542706	*PARN*	CC (344)	62.09 ± 10.09 ^a^	3	9.48 × 10^−5^	within
			TC (37)	54.82 ± 13.79 ^b^			
			TT (1)	37.60 ± NA ^b^			
	rs346121940	*PARN*	CC (344)	62.09 ± 10.09 ^a^	3	9.48 × 10^−5^	within
			TC (37)	54.82 ± 13.79 ^b^			
			TT (1)	37.60 ± NA ^b^			
	rs324883175	*PARN*	CC (344)	62.09 ± 10.09 ^a^	3	9.48 × 10^−5^	within
			AC (37)	54.82 ± 13.79 ^b^			
			AA (1)	37.60 ± NA ^b^			
	rs323242995	*SOX9*	CC (126)	63.31 ± 10.89 ^a^	12	7.88 × 10^−7^	-69369
			TC (202)	60.43 ± 10.82 ^b^			
			TT (54)	60.04 ± 9.72 ^b^			

Note: Chr denotes Chromosomes, Length indicates the relative distance (bp) between the SNP site and the candidate gene on the chromosome; “-” in the candidate gene indicates that no gene is found upstream or downstream of the site, while “within” indicates that the SNP site is located inside the candidate gene. Genotype counts may not sum to the total number of pigs per breed due to missing genotype data or quality control filtering. The ‘|’ indicates that the SNP is located in a genomic region encompassing both genes. ‘a’ and ‘b’: Different superscript letters within the same column denote significant differences (*P* < 0.05).

## Data Availability

The raw genotype presented in this study is not publicly available due to commercial confidentiality and proprietary restrictions imposed by the data provider (Guangxi Yangxiang Group Co., Ltd.), as the animals and semen samples originate from their commercial breeding program. The data that support the findings of this study are available from the corresponding author (Yunxiang Zhao, yunxiangzhao@gxu.edu.cn) upon reasonable request and with the permission of Guangxi Yangxiang Group Co., Ltd.

## Supplementary Materials

The following supporting information can be downloaded at: https://www.mdpi.com/article/10.3390/ijms27146506/s1.
