# Supplementary material for: Genome-Wide Association and Meta-Analysis Identify Candidate Genes for Sperm Freezability in Duroc and Yorkshire Boars
Source: Int J Mol Sci. 2026 Jul 22;27(14):6506. doi: 10.3390/ijms27146506 (PMC13410469; doi:10.3390/ijms27146506)
Supplement: Supplementary file 1 [file ijms-27-06506-s001.zip › Supplementary Information_ Supplementary Figures.pdf]

Supplementary Information

The following are the supplementary data related to this article:

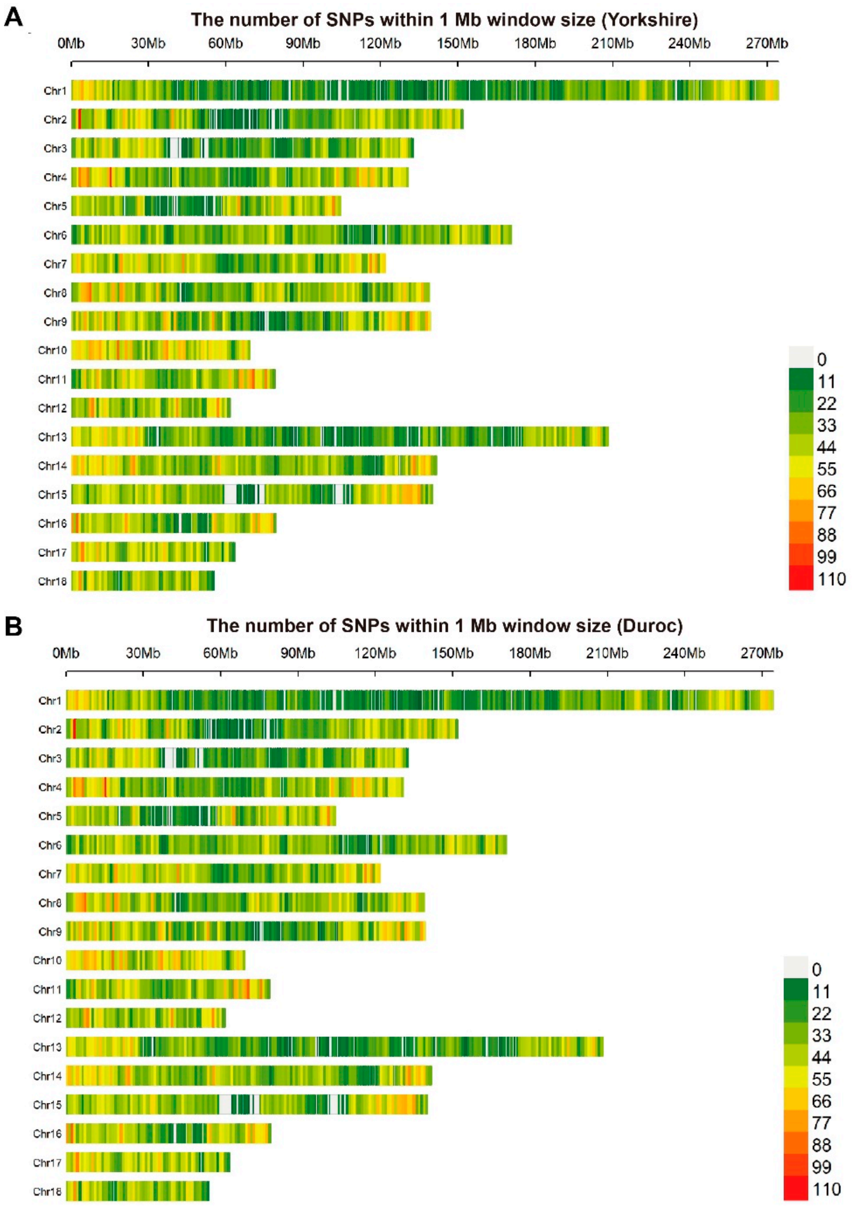

Supplementary Figure S1. Genome-wide SNP density distribution after quality control. SNP density was calculated using a 1 Mb sliding window for (**A**) Yorkshire (76,128 SNPs) and (**B**) Duroc (80,262 SNPs).

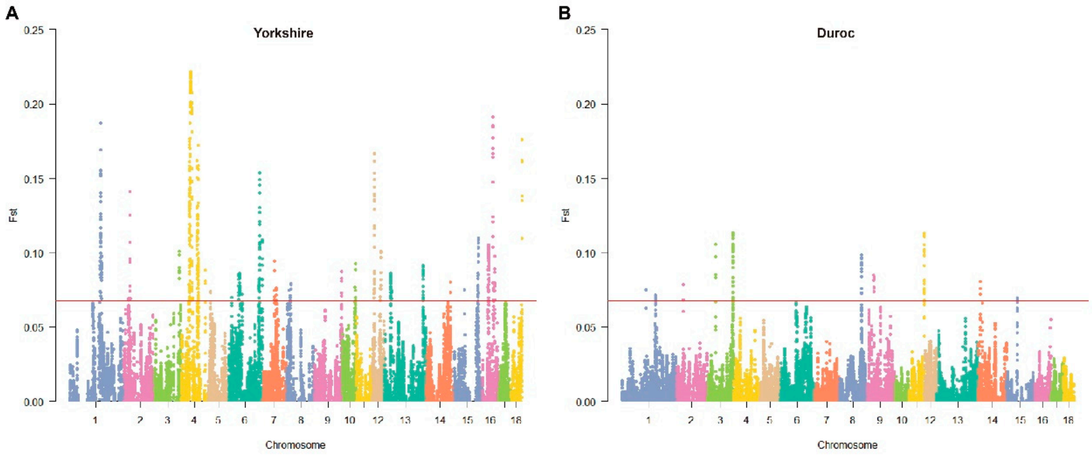

Supplementary Figure S2. Manhattan plots of genome-wide FST values between GSF and PSF groups in Yorkshire (**A**) and Duroc (**B**) boars.

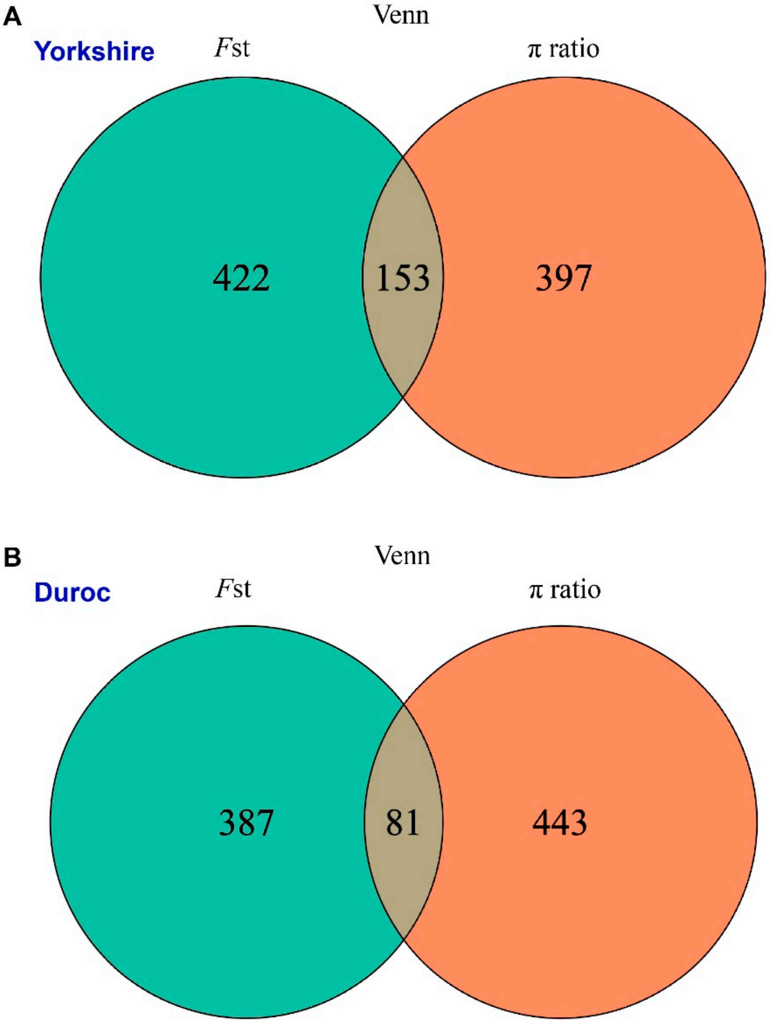

Supplementary Figure S3. Venn diagram of candidate genomic regions identified by combined F_{ST} and θ_π analysis between GSF and PSF populations in Yorkshire and Duroc boars. (**A**) Venn diagram showing the overlap of candidate genomic regions

identified by the top 1% of F_{ST} and θ_π values between GSF and PSF groups in Yorkshire boars. A total of 422 candidate regions were detected using the top 1% F_{ST} threshold ($F_{ST} > 0.082$), and 397 candidate regions were identified using the top 1% θ_π threshold ($\theta_\pi > 0.922$). The combined F_{ST} and θ_π analysis identified 153 overlapping candidate regions. (**B**) Venn diagram showing the overlap of candidate genomic regions identified by combined F_{ST} and θ_π analysis between GSF and PSF groups in Duroc boars. The overall genetic differentiation between the two groups was low ($F_{ST} = 0.0031$). The θ_π values were 0.481 and 0.488 for the GSF and PSF groups, respectively, with θ_π ratios of 0.595 and −0.588. Using the top 1% thresholds ($F_{ST} > 0.043$ and $\theta_\pi > 0.595$), 387 and 443 candidate regions were identified, respectively, with 81 regions overlapping between the two methods.

Others (See Supplementary Material):

Supplementary Table S1. Phenotypic Data.
Supplementary Table S2. Summary statistics of sperm freezability traits in Duroc and Yorkshire boars.
Supplementary Table S3. Genotype summary of 33 significant SNPs associated with sperm freezability in Yorkshire boars.
Supplementary Table S4. Genotype summary of 10 significant SNPs associated with sperm freezability in Duroc boars.
Supplementary Table S5. Meta-analysis of candidate genes associated with boar sperm freezability in boars.
Supplementary Table S6. Association analysis of variants polymorphism with sperm freezability in Yorkshire boars.
Supplementary Table S7. Association analysis of variants polymorphism with sperm freezability in Duroc boars.
